# Reconstruction of the phreno-esophageal ligament (R-PEL) prevents the intrathoracic migration (ITM) after concomitant sleeve gastrectomy and hiatal hernia repair

**DOI:** 10.1007/s00464-022-09829-z

**Published:** 2023-01-19

**Authors:** I. Hutopila, M. Ciocoiu, L. Paunescu, C. Copaescu

**Affiliations:** 1 Department of Bariatric and Metabolic Surgery, Ponderas Academic Hospital, Bucharest, Romania; 2 Titu Maiorescu University Doctoral School of Medicine, Bucharest, Romania; 3 Department of Radiology, Ponderas Academic Hospital, Bucharest, Romania; 4 Carol Davila University of Medicine and Pharmacy, Bucharest, Romania; 5 Ponderas Academic Hospital, Nicolae Caramfil Street, No. 85 A, Bucharest, Romania

**Keywords:** Laparoscopic gastric sleeve, Hiatal hernia repair, Crura approximation, Intrathoracic migration, “De novo”, GERD, Reconstruction of phreno-esophageal ligament

## Abstract

**Background:**

Laparoscopic Sleeve Gastrectomy (LSG) is the most attractive bariatric procedure, but the postoperative intrathoracic gastric migration (ITM) and “de novo” GERD are major concerns.

*The main objective* of our study was to evaluate the efficiency of the concomitant HHR with or without partial reconstruction of phreno-esophageal ligament (R-PEL) to prevent ITM after LSG. The secondary objectives focused on procedure’s metabolic and GERD-related outcomes.

**Patients and method:**

Consecutive patients who underwent primary LSG and concomitant HHR were included in a single-center prospective study. According to the HHR surgical technique, two groups were analyzed and compared: Group A included patients receiving crura approximation only and Group B patients with R-PEL. The patients’ evolution of co-morbidities, GERD symptoms, radiologic, and endoscopic details were prospectively analyzed.

**Results:**

Two hundred seventy-three patients undergoing concurrent HHR and LSG were included in the study (Group A and B, 146 and 127 patients) The mean age and BMI were 42.6 ± 11.3 and 43.4 ± 6.8 kg/m^2^. The 12-month postoperative ITM was radiologically found in more than half of the patients in Group A, while in group B, the GEJ’s position appeared normal in 91.3% of the patients, meaning that R-PEL reduced 7 times the rate of ITM. The percentage of no-improvement and “de novo” severe esophagitis (Los Angeles C) was 4 times higher in group A 3.4% vs. 0.8% with statistical significance, and correlated to ITM. The GERD symptoms were less frequent in Group B vs Group A, 21.3% vs 37%, with statistical significance. No Barrett’s esophagus and no complication were recorded in any of the patients.

**Conclusion:**

Concurrent LSG and HHR by crura approximation only has a very high rate of ITM in the first postoperative year (over 50%). R-PEL is an innovative technique which proved to be very efficient in preventing the ITM after HHR.

Laparoscopic sleeve gastrectomy (LSG) has currently become the most frequently performed surgical procedure to control the weight related morbidity [1]. To this extend, LSG addresses to a significant number of obese individuals in whom hiatal hernia (HH) and gastroesophageal reflux disease (GERD) are common findings [2, 3].

Despite its’ demonstrated metabolic efficiency, the effect of LSG to control GERD issues in bariatric patients is unclear and controversial [4]. Some studies demonstrate the LSG positive effect on GERD [5, 6] while some others argue for worsening the reflux symptoms and the related endoscopic complications [7]. No doubt, the occurrence of post-LSG gastroesophageal reflux it is a major subject due to the patients’ quality of life (QoL) impairment and the risk of possible evolution of esophagitis to Barrett’s esophagus, or even to esophageal adenocarcinoma [8].

The detailed analysis of postoperative GERD after LSG was further stimulated, and it revealed the persistence of chronic reflux symptoms or the occurrence of “de novo” GERD even with a “perfect” construction of the sleeve. Aside its multifactorial causality, the issue was mainly attributed to the postoperative HH while the need to perform hiatal hernia repair (HHR) by the time of LSG became obvious [9–11].

However, the studies aiming to analyze the GERD impact of the concomitant HHR and LSG concluded contradictory results. Some of authors found no significant difference with or without HHR [12], others GERD improvement when HHR is performed [11] while other studies demonstrate the “de novo” GERD development [13, 14].

It became clear for us that HHR is not the guaranty of GERD free after LSG and even worsening the symptoms, and the esophagitis can be an evolutive risk.

As in our institution (Ponderas Academic Hospital), a high-volume bariatric program is running, while the predominant use of LSG is following the international trend [15], we were concern about the subject. Thus, we have tried to further investigate and find out how the HHR outcomes can be improved.

A protocol of using Titanium markers for the fluoroscopic evaluation of the postoperative position of GE junction after LSG was introduced and validated with the radiologists in 2013 [16]. As a result, the presence of the radiological marker above the diaphragm was surprisingly often observed at the one-month follow-up visit during the Upper-GI X-ray study (in more than 50% of the patients) suggesting a high number of missing and unrepaired hiatal hernias by the time of LSG. The observation led to a further improvement of the surgical technique by adding a protocol of active searching for the preoperatively undiagnosed HH, in 2014 [17]. As a consequence, in our series, 43% of the patients undergoing HHR by the time of LSG were intraoperatively discovered in 2015 [16].

Even with this attitude of aggressive HHR, a high incidence of postoperative intrathoracic migration (ITM) of the upper gastric tube [18] was radiologically noted in our LSG series. To control this phenomenon, several techniques of the intra-abdominal fixation of the gastric tube were proposed, using Hill’s gastropexy [19], teres ligamentum [20], or the momentum [21].

Since 2017, we have introduced in our surgical protocol of HHR *the reconstruction of the phreno-esophageal ligament* (R-PEL) as an innovative method aiming to prevent the progressive ITM after LSG.

*The main objective* of our study was to evaluate the efficiency of the HHR with or without partial reconstruction of phreno-esophageal ligament (R-PEL) to prevent ITM after concurrent LSG. The secondary objectives focused on procedure’s metabolic and GERD-related outcomes, looking to various clinical and endoscopic correlations in the analyzed patients’ groups.

## Patients and methods

All the consecutive bariatric patients who underwent primary LSG and concomitant HHR in our center (a high-volume SRC Center of Excellence in Bariatric and Metabolic Surgery) since January 15th, 2016, were included in a prospective observational study. The Institutional Ethical Committee approved the study in 2015. As the surgical approach for HHR had modifications in 2017, the IRB was approached again, and the continuation of the study was approved. An informed consent was signed by each patient participating to the research.

In this study, the single-center cohort of LSG patients operated between January 2016 and January, 2019, was grouped according to the surgical technique used for HHR:

- ***Group A***—the patients who received HHR, defined as only crura approximation, without any esophagopexy and,
- ***Group B***—including patients with HHR to which an R-PEM was additionally performed.

*Group A* underwent surgery in the first part of the studied interval (January 15th, 2016–January 15th 2017) while *Group B* were operated later (January 15th, 2018–January 15th 2019) (Reviewer 1, Comment 2). The outcomes of the two groups were analyzed and compared.

According to the study protocol, the preoperative, intraoperative, and postoperative data of the two groups were prospectively collected and consisted of demographic characteristic, evolution of the co-morbidities, GERD symptoms, Upper-GI radiologic, and endoscopic information.

The comparative study is analyzing the 12 months’ outcomes and complications rate of the two groups.

An extensive preoperative work-up was completed in all the patients, and the indication for LSG and HHR was considered for the patients with BMI over 35 kg/m^2^, associated co-morbidities and HH. Hiatal hernia was preoperatively investigated by radiologic evaluation and endoscopy (Reviewer#2 Comment 2). The patients with long history of T2DM, non-smokers with aggressive GERD, or Barrett’s esophagus were considered for Laparoscopic RYGBP. The possible contraindications for bariatric surgery were evaluated by the multidisciplinary team.

The follow-up visits were set up at 1 month, 6 and 12 months from the surgery. For each postoperative check-up, the following parameters were assessed: weight, abdominal circumference, body mass index (BMI), excessive weight loss (EWL), and abdominal circumference (AC). The clinical evaluation looked to the presence of typical GERD symptoms (heartburn, regurgitation, epigastric pain) or nonspecific as dysphagia or vomiting. A simple symptom-specific questionnaire was completed by all the patients and documented the clinical status in the study. The questions addressed to the presence or absence of one of the typical symptoms: heartburn, regurgitation, and epigastric pain. Even assuming the subjective of the questionnaire, the GERD-positive clinical status was considered positive if any of the symptoms was present (Reviewer 1, Comment 3).

The clinical and radiological aspects were evaluated in each follow-up visit while the upper gastrointestinal endoscopy was performed only at 12 months from the intervention.

The patients who had any medical history of bariatric surgery, antireflux endoscopic or surgical procedures, as well the patients receiving associated operations (i.e., cholecystectomy, adhesiolysis) were not included in the present study. The patients who were missed at least one of the planned postoperative follow-up visits, the patients developing any type of gastric stenosis, or presenting unclear radiological images were also excluded from the study.

## Surgical technique

All the patients included in the study underwent surgery under general anesthesia with orotracheal intubation. Five to six abdominal access ports and a 45-degree 42-cm Karl STORZ endoscope (Tuttlingen, Germany) were used for LSG and HHR. For the first step of the LSG standard technique, the gastric greater curvature was dissected by means of advanced electrosurgery, using a 5 mm bipolar vessel sealer (LigaSure, Medtronic, USA) or an ultrasonic scalpel (Harmonic Ace, Ethicon, USA).

A careful inspection of the gastroesophageal junction (GEJ) and the surrounding diaphragmatic area was then performed in all the patients, following a previously described protocol [16]. This surgical step aims to confirm the endoscopic and/or radiologic findings of a hiatal hernia or to identify evidence of a preoperatively unknown HH (Reviewer#2 Comment 2).

**Fig. 1:**
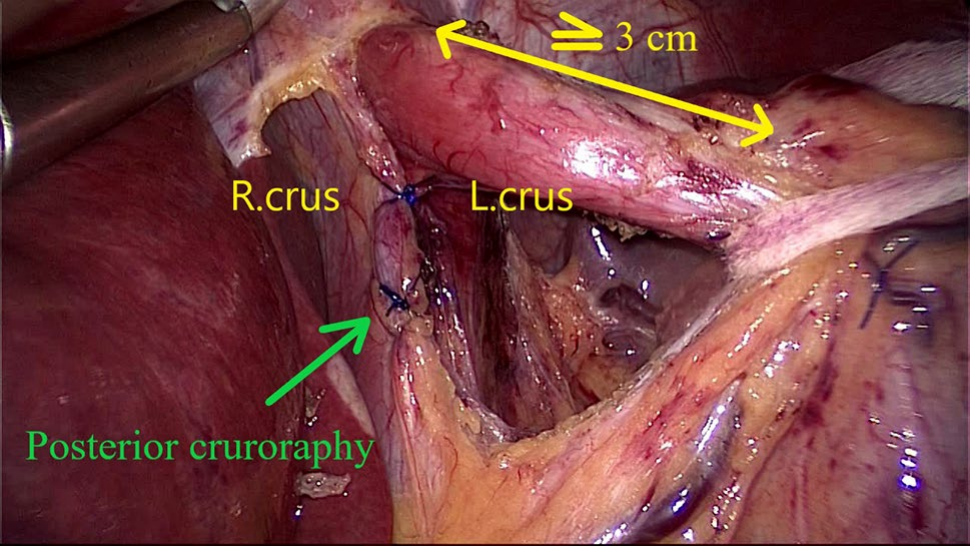
Hiatal Hernia Repair (HHR)—Crura approximation by posterior cruroraphy

The laparoscopic HHR followed two surgical steps:

1. *Dissection* initiated by the division of the *pars flaccida* and *pars condensa* of the hepato-gastric ligament, followed by adequate mobilization of the distal esophagus, to finally place cardia at least 3 cm below the diaphragm. To achieve this task, both the lower and upper leaf of the phreno-esophageal ligament (PEL) [22] are circumferentially divided. The aberrant left hepatic was preserved only if the vessels’ size was above 3 mm. The branches of the left and right vagus nerves were identified and preserved. As the result of an adequate mediastinal dissection, cardia and 3 cm of inferior esophagus should remain intraabdominally without any traction (Reviewer #1 Comment #5).
2. *Crura Approximation* As the GEJ is hooked down and to the left by a textile tie, three to four 2.0 polypropylene monofilament crossing stitches (Prolene, Ethicon, USA) were placed for the posterior cruroplasty. A 35 French bougie was used to calibrate the hiatus (Fig. 1).

**Fig. 2:**
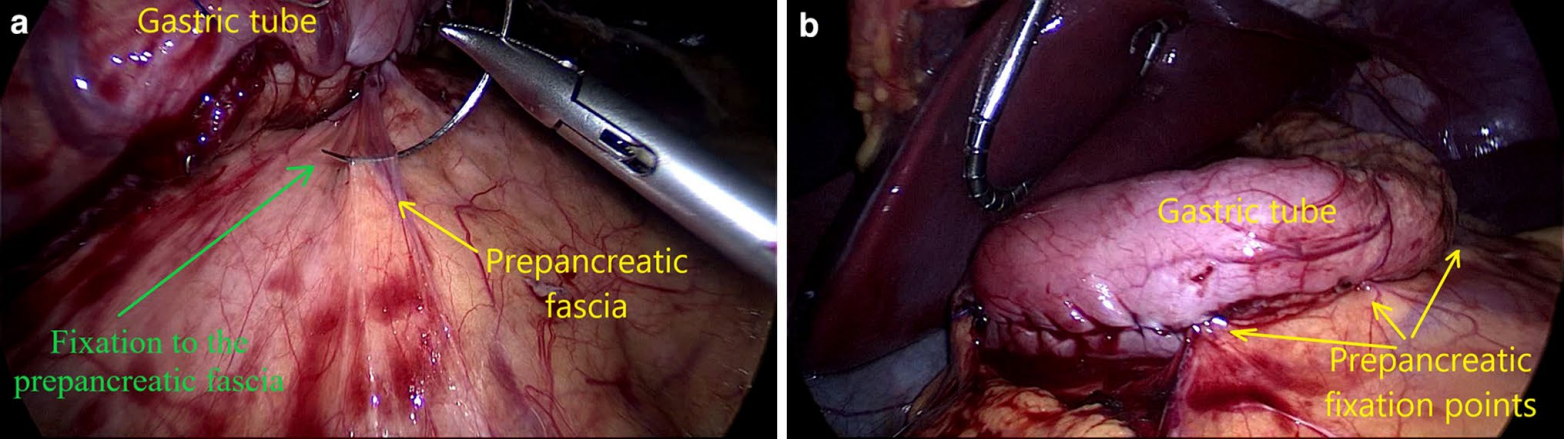
Laparoscopic Gastric Sleeve (LSG) Technique. The entire stapled line is oversewed while the gastro-pancreatic pexy (GPP) is used for the posterior fixation of the gastric tube. **a** Fixation to the pre-pancreatic fascia. **b** Final aspect

Six to eight cartridges were used for LSG, trimming up the stomach from 1 to 2 cm before the pylorus to 1 to 2 cm below the Hiss angle, along a 35 French calibration bougie. The staplers’ height was selected according to the gastric wall thickness using one of the available stapling technologies, Tri-Staple (Medtronic, USA) or Echelon GST (Ethicon, USA). Oversewing the entire stapled line using 3.0 non-resorbable monofilament suture (Prolene, Ethicon, USA) was the routine attitude. The continuous seromuscular inverting suture involved superficially the peripancreatic fascia in 2 or 3 points in all the patients included in the study (Fig. 2a). The gastro-pancreatic pexy (GPP) aims to keep the stomach in a physiologic intra-abdominal position and to prevent the twist of the long, “L-shaped,” narrow gastric tube (Fig. 2b).

**Fig. 3:**
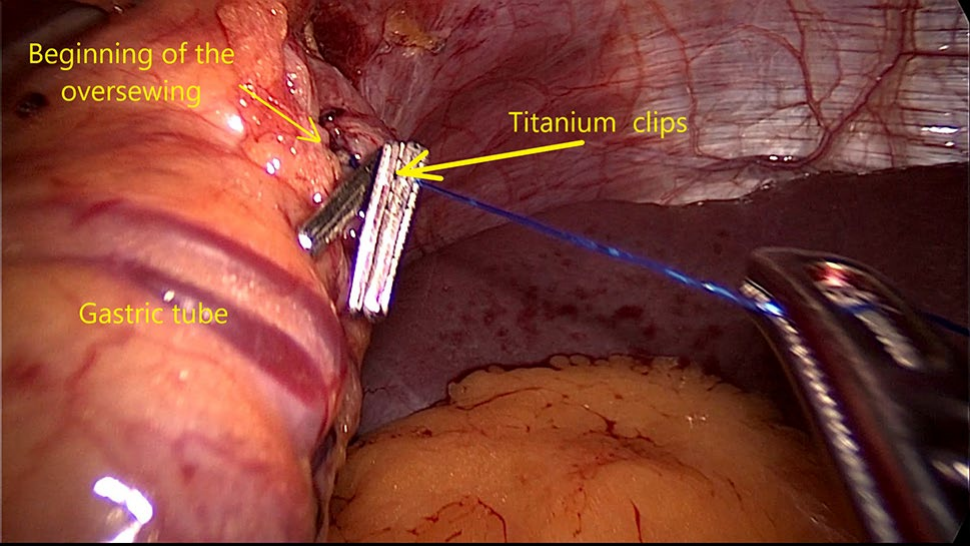
LSG technique. Titanium ML clips are applied on the polypropylene thread to radiologically mark the GEJ

Three ML Titanium Clips are applied next to each other on the proximal end of the Prolene thread to be fluoroscopically identified during the postoperative course. As the Upper-GI studies can promptly identify the radiological marker, useful information about the GEJ position relative to the diaphragm may be offered, potentially indicating a HH or a progressive ITM (Fig. 3).

**Fig. 4:**
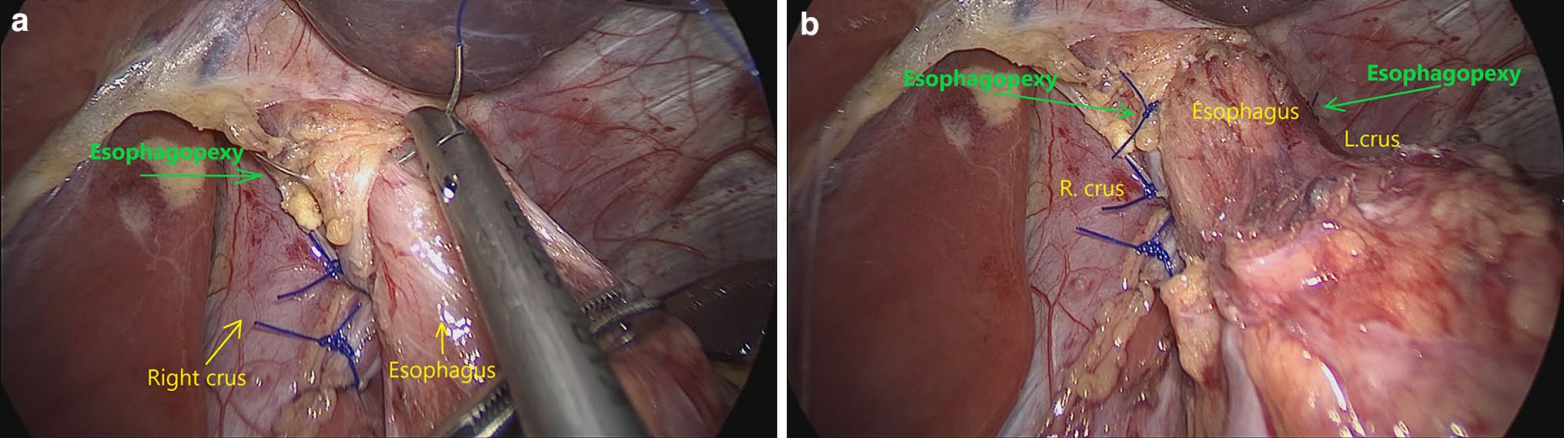
Reconstruction of the Phrenoesophageal Ligament. **a** The 3.0 polypropylene stitch is passed through the esophageal wall and the right diaphragmatic crus. **b** The two green arrows are indicating the orientation of the phrenico-esophagian pexy, at 9 and 3 o’clock (Color figure online)

For all the patients in ***Group B***, who underwent LSG and HHR since 2018, a partial *reconstruction of the phreno-esophageal ligament* (R-PEL) was added to the above-described surgical technique. To achieve this task, the esophageal wall was stitched to the surrounding diaphragm 3 cm proximal to the GEJ, at the level of original insertion of the PEL (Reviewer #1, Comment #1). Two seromuscular non-absorbable 3.0 sutures (Prolene, Ethicon, USA) were placed at 9 o’clock and 3 o’clock, on the right and, respectively, left side of the esophagus. (Figure 4a, b) aiming to create the condition to develop a connective structure between the esophagus and diaphragm, which will act as the original PEL (Reviewer 1, Comment 1).

The patients included in ***Group A***, who underwent LSG and HHR in 2016–2017, received the same surgical technique but without R-PEL. For the avoidance of doubt, both groups had the same surgical technique of LSG, including the crura approximation and the pre-pancreatic fascia gastropexy, while only the patients in Group B had R-PEL (Reviewer #1, Comment# 2).

At the end of the laparoscopic procedure, a drainage tube was routine placed in the left upper quadrant in all the patients for 24 h.

## Radiologic assessment

The protocol for radiological evaluation of the GEJ position after bariatric surgery was developed and validated by the hospital’s teams of surgeons and radiologists before initiating of this study. The methodology is based on the fluoroscopic identification of the Titanium markers placed during surgery and their position relative to the diaphragm [16].

A standard method was used for the postoperative evaluation in all the patients who underwent LSG and HHR in this study, and it is here described.

The radiological examination was performed on a digital X-ray platform, looking for a fast, simple, and reproducible way to achieve a high-quality image. All the fluoroscopies and radiographies in this study were produced on a Siemens Luminos Fusion—luorospot Compact Equipment (Siemens Healthcare GmbH, Erlangen, Germany).

The patients were asked to fast 4 h before the investigation. A total quantity of 50–100 ml barium-sulfate solution (60% w/v) was used as oral intake during each radiological examination.

The upright single-contrast barium swallow study offered evidence for the Upper-GI morphology and function. [23] Any abnormality observed at the esophagus, GEJ, gastric tube, or duodenum (i.e., stenosis, difficult passage, or contrast emptying) was mentioned.

**Fig. 5:**
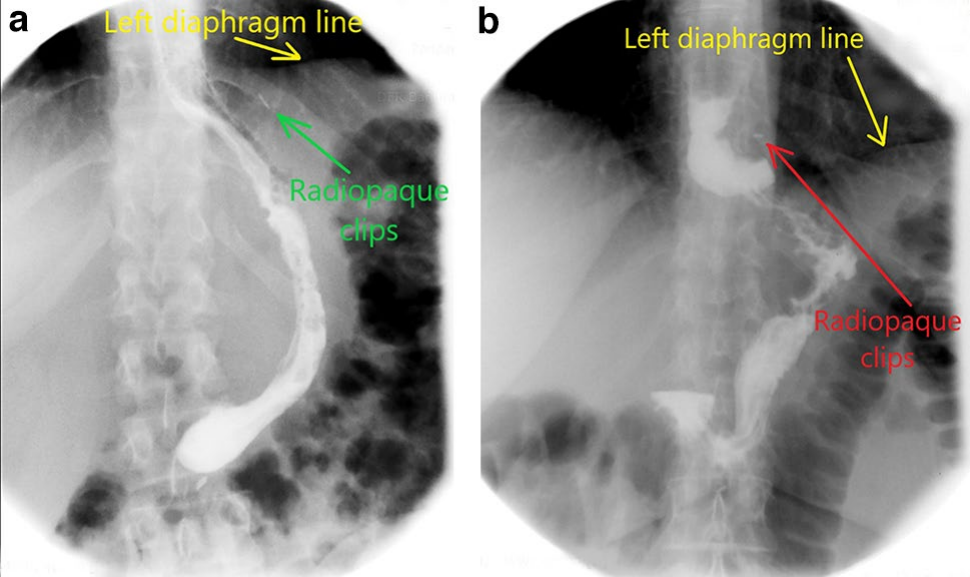
Postoperative Upper-GI study after HHR and LSG. **a** The radiologic examination demonstrates the normal postoperative position of GEJ after LSG as the radiopaque marker (Titanium Clips) is under the line of the left hemidiaphragm. **b** The radiopaque marker is above de left hemidiaphragm indicating the intrathoracic migration

The radiological assessment of the GEJ’s position was performed in standing patient, both in frontal view and right anterior oblique position (RAO), while the patient was asked to take a deep breath (*inspiration position of the diaphragm*). All the images were recorded and later independently evaluated by two radiologists and two surgeons (the authors of this paper). The changes of the GEJ’s position, relative to the diaphragm, as it was indicated by the Titanium markers placed during LSG, were dully noted. One of the following situations was considered for any of the patients in the study:

1. The GEJ is in a *normal position*—the marker placed below or at the level of the left diaphragm (Fig. 5a).
2. The GEJ and the upper gastric tube are *migrated* into the mediastinum (ITM)—the radiological marker is identified at least 1 cm above the left diaphragm (Fig. 5b). The limit of 1 cm above the diaphragm corresponds to the physiological mobility of the phreno-esophageal membrane and of the diaphragm, and it was not considered ITM) (Reviewer 1 Comment 7).

Additional information may be offered by the radiological examination in patients with ITM like the aspect of a tortuous esophagus, esophageal dilatation, or contrast-emptying impairment.

The patients for whom the images were unclear or confuse were excluded from the study.

## Upper gastrointestinal endoscopy

**Fig. 6:**
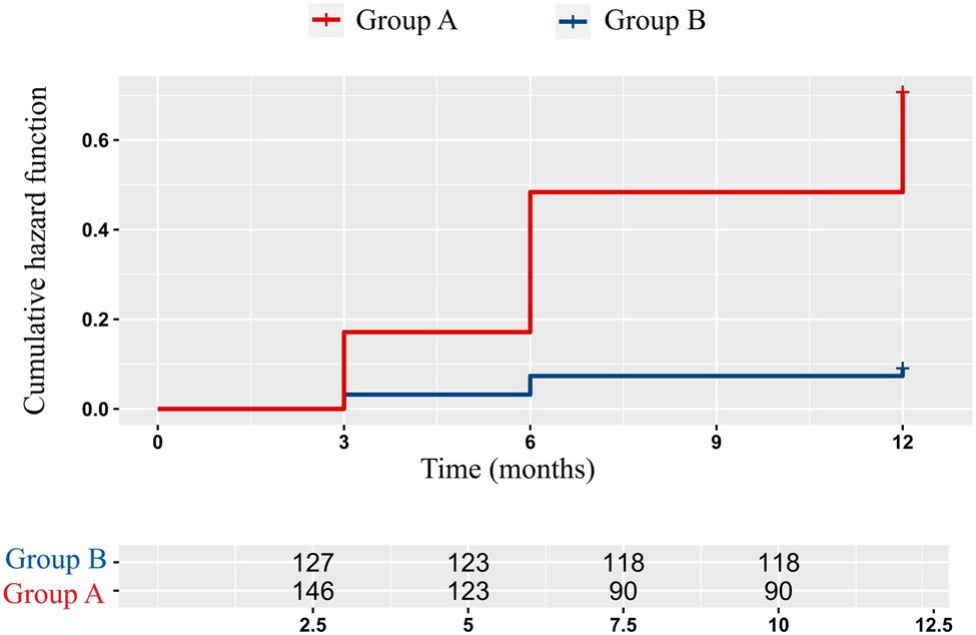
Cumulative hazard risk for ITM

Esophagogastroduodenoscopy (OGD) was performed preoperatively and 12 months after surgery in all the patients included in the study. The patients underwent OGD under intravenous sedation with benzodiazepines (Midazolam 1 mg/ml) or Propofol [24]. An anesthetist was always present for OGD when Propofol was used. The Olympus Exera III Endoscopic System (Olympus, Japan) was used for the endoscopic investigations (Fig. 6).

The presence of the HH and any other morphologic or functional Upper-GI pathological aspect were noted by the endoscopist. Esophagitis was scored according Los Angeles classification [25]. Multiple biopsies were taken in all the patients presenting endoscopic aspects suspected for Barrett’s esophagus [26].

The OGD role to identify HH after LSG is challenging due to an often-modified distal esophagus, an unclear positioning of the Z—line relative to the diaphragmatic hiatus while the “U-turn” should be impossible in a correct LSG.

If any postoperative complication required OGD after LSG and HHR (i.e., stenosis, bleeding, etc.), the patient was excluded from the study.

The OGD was performed by any of the nine endoscopists of the Center and the reports were assumed in the study as they were concluded by the physician on duty. No audit or second validation of the recorded endoscopic images was performed. Besides this, no preoperative or postoperative 24 pH monitoring evaluation was performed for the patients included in the study.

As consequence, to avoid ambiguities of a subjective interpretation of the endoscopic aspect, we have decided to look at and limit only to the evolution of severe esophagitis (Los Angeles C) and Barrett’s esophagus as these aspects were described by the OGD.

### Statistical analysis

Data were analyzed using the R project software, version 4.0.2. Copyright (C) 2020. The R Foundation for Statistical Computing, R Core Team (2020), Vienna, Austria. URL https://www.R-project.org.

A Kaplan—Meier method was used to analyze time-to-event (gastric migration) data and a Cox-proportional hazards regression with Aalen additive models for variables that influence the occurrence of migration.

Data were presented as mean ± SD for continuous variables and absolute frequency and relative frequency for the categorical variables. A *p* value less than 0.05 was considered representative for statistical significance.

## Results

**Fig. 7:**
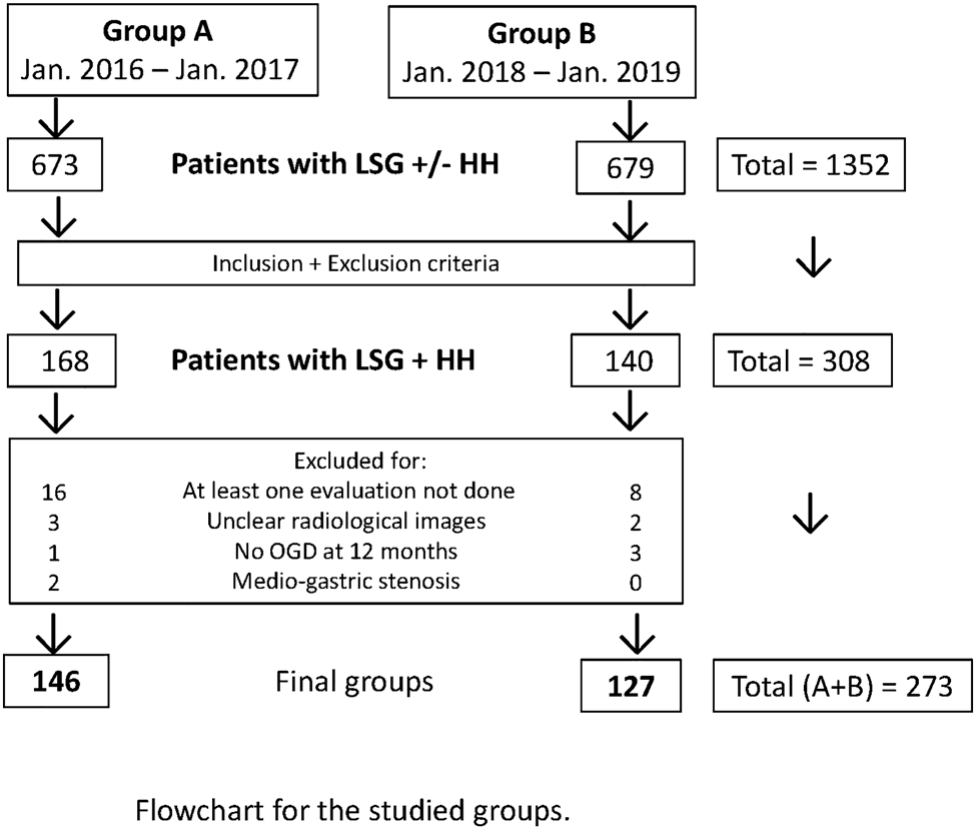
The flowchart of the groups analyzed in the study, selected according to the including and excluding criteria. *LSG* laparoscopic sleeve gastrectomy, *HH* hiatal hernia, *OGD* oesophago-gastro-duodenoscopy

Three hundred eight patients who fulfilled the including criteria were registered in the prospective single-center observational study while a total of 1352 patients received LSG during the analyzed time intervals. (Flowchart—Fig. 7) (Reviewer#1 Comment 4) (Reviewer #2 Comment 1). Thirty-five patients were excluded from the study as they missed at least one postoperative evaluation (24 patients), had unclear radiological images (5 patients), because the 12-month OGD was not performed (4 patients) and because they developed a medio-gastric stenosis (2 patients, later successfully treated by several endoscopic dilatations) (Reviewer# 2 Comment 4).

A total number of 273 patients (95 male and 178 female) with a mean ± SD age of 42.6 ± 11.3 and a mean BMI of 43.4 ± 6.8 (Reviewer# 1 Comment 6) were included in the comparative study.

One hundred forty-six patients who underwent LSG and HHR were selected for the Group A and 127 patients in whom R-PEL was additionally performed were included in the Group B. No mesh was used for HHR in any of the patients in this study.

The patients’ demographics, preoperative characteristics and co-morbidities are presented in Table 1. The data analysis demonstrated that there are no significative statistical differences between the two groups.

**Table 1:** Preoperative variables for the study groups

	GroupA (*N* = 146)	GroupB (*N* = 127)	*P* value
Age			0.481
Mean (SD)	42.3 (10.6)	42.9 (12.0)	
Median [Min, Max]	42.0 [18.0, 65.0]	44.0 [18.0, 65.0]	
Sex			0.961
F	95 (65.1%)	83 (65.4%)	
M	51 (34.9%)	44 (34.6%)	
AC			0.835
Mean (SD)	130 (15.7)	129 (15.1)	
Median [Min, Max]	129 [98.0, 188]	129 [90.0, 165]	
BMI (kg/m^2^)			0.343
Mean (SD)	43.2 (7.21)	43.6 (6.30)	
Median [Min, Max]	41.9 [35.0, 80.5]	42.3 [35.0, 66.0]	
GERD symptoms			0.117
No	94 (64.4%)	93 (73.2%)	
Yes	52 (35.6%)	34 (26.8%)	
HTN			
No	73 (50.0%)	65 (51.2%)	0.846
Yes	73 (50.0%)	62 (48.8%)	
T2DM			0.094
No	129 (88.4%)	103 (81.1%)	
Yes	17 (11.6%)	24 (18.9%)	
Dyslipidemia			0.005
No	20 (13.7%)	5 (3.9%)	
Yes	126 (86.3%)	122 (96.1%)	
OSA			
No	106 (72.6%)	83 (65.4%)	0.196
Yes	40 (27.4%)	44 (34.6%)	
Esophagitis			0.7
No	89 (61.0%)	75 (59.1%)	
Mild (A + B)	54 (37.0%)	50 (39.4%)	
C	3 (2%)	2 (1.6%)	

*AC* abdominal circumference, *BMI* body mass index, *HTN* arterial hypertension, *GERD* gastroesophageal reflux disease, *OSA* obstructive sleep apnea, *T2DM* type 2 diabetes mellitus

Preoperative GERD symptoms and severe esophagitis were similarly present in both groups of bariatric surgery candidates, in 35.6% vs 26.8% and 2% vs 1.6%, respectively. To be noted that most of the patients were free of GERD symptoms and endoscopically no or mild esophagitis was mentioned in the majority of both groups, despite the certified presence of the HH which was subjected for repair.

The metabolic surgery efficiency was demonstrated in both groups. Similar positive evolution of the AC, BMI, and EWL% was noted 1 year after the surgery in Group A and in Group B, and data are presented in Table 2.

**Table 2:** Postoperative variables for the study groups

	GroupA (*N* = 146)	GroupB (*N* = 127)	*P* value
AC Postop 1 year			1
Mean (SD)	93.7 (12.9)	93.7 (13.1)	
Median [Min, Max]	92.5 [67.0, 141]	94.0 [62.0, 130]	
AC Diff. 1 year			0.4
Mean (SD)	36.4 (11.4)	35.3 (11.1)	
Median [Min, Max]	35.0 [17.0, 68.0]	35.0 [9.00, 61.0]	
BMI Postop. 1 year			0.86
Mean (SD)	27.6 (5.11)	27.5 (4.47)	
Median [Min, Max]	26.5 [19.6, 56.0]	27.0 [19.0, 43.0]	
EWL% 1 year			0.87
Mean (SD)	86.7 (15.6)	86.4 (16.2)	
Median [Min, Max]	90.0 [45.0, 140]	87.2 [47.5, 125]	
Esophagitis postop 1 year			0.2
No	64 (43.8%)	74 (58.2%)	
Mild (A + B)	77 (52.7%)	52 (41%)	
C	5 (3.4%)	1 (0.8%)	
ITM			0.01
No	72 (49.3%)	116 (91.3%)	
Yes	74 (50.7%)	11 (8.7%)	
GERD 1 year			0.01
No	92 (63.0%)	100 (78.7%)	
Yes	54 (37.0%)	27 (21.3%)	

*AC* abdominal circumference, *AC Diff.* abdominal circumference difference at 1 year (AC preop.—AC at 1 year), *BMI* body mass index, *%EWL* excess weight loss, *HTN* arterial hypertension, *GERD* gastroesophageal reflux disease, *ITM* intrathoracic migration, *OSA* obstructive sleep apnea, *T2DM* type 2 diabetes mellitus

The radiological evaluation of the GEJ, 12 months after LSG and HHR indicated ITM in more than half of the patients who underwent crura approximation only (group A) while in the Group B, the normal position of the gastric tube was demonstrated in 91.3% of the patients. This evidence clearly indicates that partial R-PEL was efficient, as the number of the patients developing recurrent HH or ITM 1 year after surgery was almost 7 times higher in the Group A compared to the Group B. The R-PEL failed to prevent ITM in some patients (8.7%), and this can be explained by the early breaking of the esophageal pexy points.

Further analysis of the 1-year postoperative data showed more cases of severe esophagitis (Los Angeles C) in group A (5, 3.4%) and only one in Group B. No cases of Barrett’s esophagus were identified at 1 year postoperatively in any of the groups**.**

A detailed analysis of the very patients with severe esophagitis demonstrated that, in the Group B, the two patients who preoperatively had severe endoscopic GERD had no esophagitis 1 year after HHR and R-PEL. However, one patient in this group presented “de novo” severe GERD 12 month after surgery, and he was in the subgroup B positive for ITM.

Same analysis of the patients with severe esophagitis in Group A indicated that the preoperative severe GERD (2%) was not healed after HHR while “de novo” aggressive GERD was founded in two other patients. All these 5 patients were identified in the subgroup A of positive postoperative ITM.

The analysis of the clinical signs showed more *GERD-symptom free patients* 1 year after the surgery in Group B (78.7%) as compared with Group A (63.0%). The difference is statistically significant, again demonstrating the efficiency of the R-PEL for the HHR concomitant with LSG.

Moreover, if these results are compared to the preoperative ones, GERD was improved in the Group B (78.7% vs 72.3%) and slightly worsened for the patients in Group A (63.0% vs 64.4%). Even without statistically significant differences, the data suggest the potential efficiency of HHR and R-PEL to control GERD symptoms in LSG patients.

The multivariate analysis of the progressive thoracic migration of the gastric tube over the first postoperative year is depicted in Fig. 7. An approximately 11 times higher probability (odds) of the hiatal hernia recurrence at 1 year was noted for the patients in Group A as compared with Group B, and the effect is statistically significant (*p* < 0.01). The average period of hernia recurrence was more than 2 months shorter for patients in Group A than for patients in Group B.

No intraoperative or postoperative complications, nor deaths were encountered in any of the patients included in the study.

No complications or adverse effects have been recorded for any of the radiologic or endoscopic investigations performed in the study.

No surgical or endoscopic interventions for severe GERD, esophagitis, or HH recurrence were performed in any of the participants before the end of the study**.**

## Discussions

Bariatric surgery (BS) has proved its efficiency for the morbid obesity [4], but the metabolic affected population is increasing very fast [27] challenging the surgical offer. The introduction of laparoscopic sleeve gastrectomy (LSG) on a large scale extended the BS offer [28], while lowering the risk of postoperative complications [29]; therefore, it became rapidly the first option in the surgeons and patients preference [1].

On the other hand, obesity is associated with high incidence GERD, as high as 70% [30], while in bariatric surgery candidates, HH and GERD were preoperatively present in 40% [2] and over 60% [3] of the cases, respectively. To this extend, the more frequently performed procedures of LSG had to face this GERD problem, too. As LSG was considered a “high pressure system” [31], it has been initially limited for patients with GERD, Roux en Y gastric bypass (RYGBP) being recommended in those situation [32].

Nevertheless, after improving the surgical performance of LSG and carefully avoiding the technical issues (obstructions, twists or narrowing of the gastric tube), positive effects on GERD were noticed after this procedure [5, 6], hence, explained by the reduced intra-abdominal pressure after weight loss, a reduced acid production, and the accelerated gastric emptying [33, 34].

Moreover, if HHR is performed by the time of LSG, a good control of GERD is noticed and the “de novo” reflux is uncommon [35]. To support this, the systematic review published by Mahawar et al. showed favorable results in 16 of 17 studies comparing the effects of simultaneous sleeve gastrectomy and hiatus hernia repair on GERD [36].

On the contrary, other studies argue for worsening the reflux symptoms and the related endoscopic complications, identifying “de novo” GERD and Barrett’s after LSG, introducing the fear of potential evolution to esophageal adenocarcinoma in these patients [7, 8, 37, 38].

Besides the multifactorial development of postoperative GERD, a common explanation for the presence of these complications even after a “perfect sleeve” is based on the fact that HH and GERD are not properly identified during the preoperative work-up and yet not addressed during LSG. And this fact may not surprise us, as clinical and endoscopic correlations for GERD are very weak in obese patients [39]. Consequently, concurrent LSG and HHR is performed less frequent than the above-mentioned expected presence, ranging from 2.4 to 18,5% [40–42].

In our hospital (CoEBMS), since we start to apply the described protocol of radiological examination during the follow-up Upper-GI studies in all the patients receiving LSG, we have observed that the Titanium marked GEJ has a constant tendency to move cranially, as part of a hiatal hernia or an intrathoracic migration (ITM). As a consequence, a protocol of intraoperative active search for HH during bariatric surgery was introduced in 2014 in our current practice [17]. As a result, more HH were identified and, as high as 43% of the total hiatal hernias repaired concomitant with LSG were intraoperatively discovered [16]. The technique use for HHR was similar which the one widely recommended during LSG, meaning the complete dissection and abdominal mobilization of the GEJ and of the inferior esophagus followed by crura approximation with non-resorbable 2.0 stiches [42]. All the patients included in Group A of the present study underwent concomitant LSG and HHR using this technique.

But, as we continued to use the above-mentioned radiological protocol, even after increasing the rate of HHR, we continued to frequently observe the radiological marker above the diaphragm, meaning that the HHR is unable to control the migration phenomenon. Moreover, other studies have demonstrated the persistence of the reflux symptoms and “de novo” GERD after LSG regardless the HHR and the possible explanation was the upward migration of the gastric tube [8, 43].

A fundamental question had to be addressed for the future of this bariatric procedure: is it the LSG concept or the HHR technique responsible for the “de novo” GERD and uncontrolled phenomenon of ITM?

Looking for answers, we have noticed that the “migration crisis” is affecting other bariatric procedures, too. Arnoldner et al. studied prospectively thirty patients who underwent RYGBP and discovered CT evidence for ITM in 66.7% of them, whereas gastroscopy did not correctly identify any herniation and symptomatic reflux in one third of the patients [44].

On the other hand, as only a minority of the bariatric patients experience GERD and ITM after LSG, the responsibility may not be on the concept of LSG but rather on HHR.

**Fig. 8:**
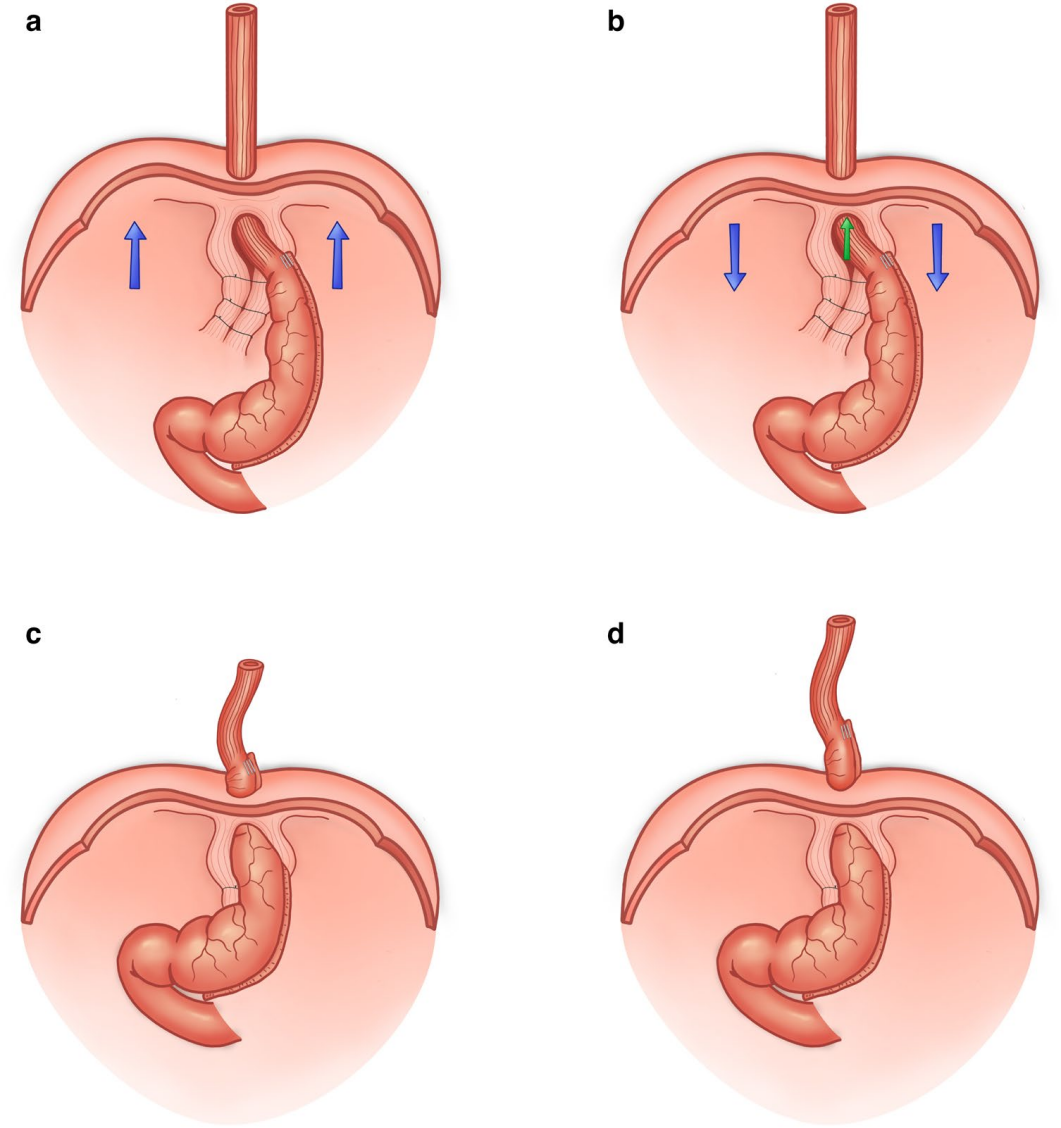
The scenario of ITM after concurrent LSG and HHR. **a** CO_2_ pneumoperitoneum is lifting the diaphragm while, at the time of finalizing the crura approximation, the GEJ is left below the diaphragm. **b** as the CO_2_ exsufflation starts, the diaphragm moves down and the GEJ slides up (ITM). **c** ITM continues during the early postoperative course until the postoperative adherences are bonding the anatomical structures. **d** The process of ITM may continue during the following years being responsible for all the consequences of the GERD after a migrated LSG

Further analyzing the technique of HHR, one may notice that, during the dissection steps, the phreno-esophageal ligament (PEL) is circumferentially divided to allow the proper mobilization of the GEJ. One of the authors of these study (Catalin Copaescu) observed that, after crura approximation, the GEJ may easily slide up into the posterior mediastinum as no substitute for PEL is in place. This was a fundamental observation for this research and the hypothetic scenario is presented in Fig. 8. In fact, during laparoscopy, the CO_2_ pneumoperitoneum is lifting the abdominal wall and the diaphragm, too. By the time of finalizing the proper crura approximation, the GEJ is left 3–4 cm below the diaphragm and the surgeons are expecting to permanently remain in this position (Fig. 8a). However, as the CO_2_ exsufflation starts, the diaphragm moves down and the GEJ slides up, minutes after the surgery (Fig. 8b). The process continues during the early postoperative course until the postoperative adherences are bonding the anatomical structures. By the time of complete local healing after HHR, the GEJ may be already high in the mediastinum (Fig. 8c). In other words, the migration phenomenon is only a question of time after LSG unless a method for maintain the GEJ in the abdomen is used. The process of ITM may continue during the following years being responsible for all the consequences of the GERD after a migrated LSG (Fig. 8d).

The phreno-esophageal ligament (PEL) is composed of abundant collagen and elastic lamellae, and it is bridging the space between the esophageal wall and the margins of the esophageal hiatus. This ligament appears to arise from both the endothoracic fascia and the transversalis fascia and, on its way toward the esophagus it divides into an upper and an lower leaf before inserting deep into the esophageal wall [45]. PEL plays an important role in anchoring the lower esophagus and maintaining gastroesophageal competence [22].

By dividing PEL during HHR, its role is totally suspended and, we may not be surprised by the progressive consequences.

Sader et al. clearly demonstrated in an experimental comparative study that, dividing the PEL, a perfect condition for developing huge sliding hiatal hernias is created [46]. In two other groups of dogs analyzed in this study, the esophageal hiatus was narrowed and, either an artificial PEL, made of a wide strip of synthetic material or two retroesophageal stitches were additionally used. Both methods were efficiently preventing the HH recurrence after opening the hiatus [46].

In our study, we have introduced the concept of reconstruction of the phreno-esophageal ligament (R-PEL) aiming to maintain LES and GEJ in the abdomen, accomplishing the first principle of antireflux surgery [47]. To achieve this, two opposite stitches are used to create a phreno-esophageal bridging at 3–4 cm above cardia. This pexy intends to limit the early postoperative cranially sliding of the GEJ and of the gastric tube, and it may further orient the extension of the endothoracic and transversalis fascia toward the esophagus during the healing process. Finally, a scarring structure inserted similarly to the original one will be developed, anchoring the lower esophagus into the abdomen, and preventing the ITM.

All the patients in Group B received LSG, HHR, and R-PEL and the probability to develop ITM within the first postoperative 12 months has been reduced 11 times(odds) as compared to the patients in Group A for whom no substitute for PEL but only HHR by crura approximation was used.

The endoscopic examination demonstrated improvement of the GERD complications as the ITM could be prevented. This is also proving the fact that the sleeve concept is not refluxogenic but the gastric tube migration might be mostly responsible for postoperative GERD. However, the study protocol was based and limited to the endoscopist’s observation and reports while no correlations with the 24 h pH monitoring studies were performed in the analyzed groups.

Further analyzing the results of the study, we could demonstrate that R-PEL is superior to gastro-pancreatic pexy (GPP) in preventing ITM. GPP was introduced in our current practice in 2014, aiming to reduce the axial twist after LSG but we have also expected that by attaching the stomach to a fixed retroperitoneal structure (the pancreas), the gastric tube may be maintained in the abdomen. The first objective was reached [48] but the ITM was not controlled. All the patients in the Group A had GPP only and the ITM rate 1 year later was present in more than half of the patients.

Rather than GPP, two other types of gastropexy were proposed to control the reflux complications after LSG. One of them is the stapled line omentopexy, [49] but it did not have a significant effect on reducing the incidence of de novo GERD after LSG [50]. These limitations may be explained by the fact that the stomach is attached to a relatively mobile structure (the ommentum), thus, being unable to limit the ITM.

A modified Hill’s gastropexy was also proposed as a possible surgical technique to control GERD after LSG [19] but the literature scarce for the outcomes of these procedures. Sánchez-Pernaute et al., presented good 6 months results in one patient [19] and Nassar et al. observed satisfactory GERD control in a 3-year retrospective study including 16 patients [51]. Nevertheless, stitching cardia to the preaortic fascia may narrow the GEJ and cause postoperative dysphagia [47]. We consider R-PEL superior as it is keeping the LES into the abdomen using more bridging points to the diaphragmatic hiatal contour, thus, preventing ITM while leaving cardia free, avoiding postoperative dysphagia.

We expect some criticism for R-PEL as, the classic principles of antireflux (AR) are excluding any stitching of the esophagus to the diaphragm, leaving the two structures to move independently [47]. Indeed, the concept of AR includes crura approximation and a 360° or partial fundoplication, acting to keep the GEJ in the abdomen while the diameter of the gastric wrap is large enough to prevent the ITM. But, as PEL is a physiologic presence, and it plays a significant role in maintaining GEJ in the abdomen, thus, preventing the ITM [45, 46] bridging the esophagus to the diaphragm should not be restricted anymore. Furthermore, a fundoplication is not facile in the case of LSG or any gastric bypass.

Several techniques of fundoplication-like have been recently introduced to control the GERD issues after LSG: Nissen Sleeve (N-sleeve) [52], anterior (Dor) fundoplication and sleeve (D-Sleeve) [53], Rossetti-Sleeve (R-sleeve) [54], and Toupet Sleeve [55]. None of them are acting in the classical meaning of AR surgery as the fundus wrap is small and stretched, almost excluded from the rest of the stomach, while offering a very limited room to work as an AR pneumo-valve [47]. Their positive effect on GERD after LSG may be related to the physical presence of the gastric wrap blocking the GEJ below the diaphragm. We consider that R-PEL is simpler, more efficient and safer, avoiding the possible postoperative complications. Carandina et al. reviewed the literature looking to the outcomes of different fundoplication associated with LSG, and they found an overall postoperative complication rate of 9.4%, mostly gastric perforations (3.1%), bleeding (1.8%), and gastric stenosis (1.2%) [56] Moreover, like any other fundoplication, all these newly introduced procedures may be in time associated with intrathoracic migration [57]. Hainaux et al. found that, in the first postoperative day, the intact Nissen fundoplication migrated cranially in 30% of the patients [57].

The ligamentum teres cardiopexy (Narbona-Arnau procedure) was proposed to prevent acid reflux by reinforcing the lower esophageal sphincter and restoring its competence in patients with previous sleeve gastrectomy and hiatal hernia [20]. However, the R-PEL is less complex, saving the time of harvesting the teres ligamentum and avoiding its’ delicate wrapping around the esophagus, carrying a high risk of dysphagia [58] We prefer the Narbona-Arnau procedure for the recurrent ITM after LSG or gastric bypass [59], R-PEL being our first options for primary HHR.

The technique of R-PEL proved to be safe in our study. No intraoperative or postoperative complication related to the procedure has been recorded. However, care should be taken to consistently involve in the suture both the diaphragmatic hiatal border and the muscular layers of the esophagus, to avoid penetrating the esophageal lumen, any laceration of the esophageal wall or knotting too loose.

Nevertheless, gastric stenosis was encountered in the group of patients who underwent LSG and HHR only, and the complication was solved by endoscopic dilatation. According to the study’s protocol, they were excluded from the research as they may be responsible for the postoperative GERD.

The proposed technique was not using any synthetic material or mesh to reconstruct the PEL, thus avoiding the possible foreign body related complications.

However, the R-PEL failed to prevent ITM in some patients (8.7%), and this can be explained by the early breaking of the esophageal pexy points, as only two stitches were used in the surgical protocol and some of the patients experienced transitory episodes of vomiting during the early postoperative course. Since the failure mechanism has been understood, the R-PEL was improved. Starting with 2020, not anymore two but three stitches were placed symmetrically at 10, 2 and 6 o’clock to orient the R-PEL (Reviwer#3 Comment 4).

## Strength and limits of the study

To our knowledge, this is the largest single-center prospective study analyzing the outcomes of the concurrent HHR and LSG, focusing on ITM. An accessible and efficient protocol of upper GI radiologic evaluation of postoperative migration after bariatric surgery has been introduced, enlarging the limits of the CT, MRI, or OGD to study the migration phenomenon. The reconstruction of phreno-esophageal ligament is here proposed as an innovative, safe, efficient, and relatively simple method to prevent and control the ITM after LSG. The simple revision of gastric sleeve to RYGBP for reflux issues is not a guaranty for the GERD and ITM control [44] R-PEL may be successfully and efficiently used for other types of bariatric procedures, such as gastric bypass also at the risk of ITM. The potential mechanism of postoperative ITM has been clearly described in this paper while R-PEL may be a revolutionary concept aiming to improve the antireflux surgery and prevent some of the long-term complications.

R-PEL proved to be superior in preventing postoperative ITM after LSG as compared with any other gastropexies. However, comparable studies RCT with other methods of fixation are needed.

The limits of our study are related to the sample size, the time of follow-up (1 year) and no correlation with 24 h pH monitoring (Reviwer#3 Comment 4). Moreover, the wide use of the described radiologic evaluation may be limited as fluoroscopy is not easily accessible in some centers. The study protocol used two radiologist and two surgeons to validate the results but, currently only one radiologist or surgeon may easily notice the stage of ITM following the position of the titanium marked GEJ on the postoperative images after BS. Nevertheless, the accuracy of our radiologic study might be validated by comparative CT/MRI examinations. Furthermore, extensive multicentric and RCT studies are needed to verify the results of the present research.

## Conclusion

Concurrent sleeve gastrectomy and hiatal hernia repair by cruroplasty only have a very high probability of intrathoracic migration in the first postoperative year (over 50%).

The reconstruction of phreno-esophageal ligament is proposed as an innovative, safe, efficient, and relatively simple method to prevent and control the ITM after LSG.

R-PEL is not preventing the ITM after LSG as the HH was not identified and yet repaired. The results of the present study suggest that GERD after LSG is not in the responsibility of the concept of sleeve but related to other additional surgical procedures that might improve the outcomes, such as HHR and R-PEL.

Nevertheless, longer follow-up and comparative multicentric and RCT studies are needed to confirm the role of reconstruction of PEL in prevention of ITM after LSG + HHR and its applicability for other procedures at the risk of ITM.

## Abbreviations

AC: Abdominal circumference
BMI: Body Mass Index
CT: Computed tomography
T2DM: Type 2 diabetes mellitus
EWL: Excess weight loss
GERD: Gastroesophageal reflux disease
HTN: Hypertension
HH: Hiatal hernia
ITM: Intrathoracic gastric migration
HHR: Hiatal hernia repair
LSG: Laparoscopic sleeve gastrectomy
OGD: Eesophago-gastro-duodenoscopy
